# Monitoring protein turnover during phosphate starvation-dependent autophagic degradation using a photoconvertible fluorescent protein aggregate in tobacco BY-2 cells

**DOI:** 10.3389/fpls.2014.00172

**Published:** 2014-04-30

**Authors:** Maiko Tasaki, Satoru Asatsuma, Ken Matsuoka

**Affiliations:** ^1^Graduate School of Bioscience and Biotechnology, Kyushu UniversityFukuoka, Japan; ^2^Faculty of Agriculture, Kyushu UniversityFukuoka, Japan; ^3^Biotron Application Center, Kyushu UniversityFukuoka, Japan; ^4^Research Center for Organelle Homeostasis, Kyushu UniversityFukuoka, Japan

**Keywords:** autophagy, phosphate, phosphite, photoconvertible fluorescent protein, tobacco BY-2 cells, chase experiment, protein aggregate, turnover

## Abstract

We have developed a system for quantitative monitoring of autophagic degradation in transformed tobacco BY-2 cells using an aggregate-prone protein comprised of cytochrome b5 (Cyt b5) and a tetrameric red fluorescent protein (RFP). Unfortunately, this system is of limited use for monitoring the kinetics of autophagic degradation because the proteins synthesized before and after induction of autophagy cannot be distinguished. To overcome this problem, we developed a system using kikume green-red (KikGR), a photoconvertible and tetrameric fluorescent protein that changes its fluorescence from green to red upon irradiation with purple light. Using the fusion protein of Cyt b5 and KikGR together with a method for the bulk conversion of KikGR, which we had previously used to convert the Golgi-localized monomeric KikGR fusion protein, we were able to monitor both the growth and *de novo* formation of aggregates. Using this system, we found that tobacco cells do not cease protein synthesis under conditions of phosphate (Pi)-starvation. Induction of autophagy under Pi-starvation, but not under sugar- or nitrogen-starvation, was specifically inhibited by phosphite, which is an analog of Pi with a different oxidation number. Therefore, the mechanism by which BY-2 cells can sense Pi-starvation and induce autophagy does not involve sensing a general decrease in energy supply and a specific Pi sensor might be involved in the induction of autophagy under Pi-starvation.

## Introduction

Plants have various ways of responding to limitations in their nutrient supply to ensure their survival. One such response is the induction of autophagy, which is the digestion by a cell of its own intracellular contents. It has been shown that this reaction occurs at the cellular level because autophagy can be induced in cultured cells by changing the regular medium to a nutrient-deficient one. In the case of tobacco BY-2 cells, autophagy is induced when there are restricted supplies of sugar, nitrogen, or phosphate (Pi) in the medium (Moriyasu and Ohsumi, 1996; Toyooka et al., 2006).

The *in planta* responses to Pi-starvation have been linked to sugar-signaling pathways (Rouached et al., 2010). In contrast to the *in planta* responses that require days after exposure to Pi-starvation, the induction of autophagy of BY-2 cells under nutrient-depleted conditions including Pi-starvation requires less than 12 h (Toyooka et al., 2006). The loss of Pi in the medium not only induces autophagy (Toyooka et al., 2006), but also causes cell cycle arrest of BY-2 cells at the G1 phase (Sano et al., 2004). Thus, information regarding a low extracellular level of Pi is transmitted not only to induce autophagy, but also to prevent progression of the cell cycle. In contrast to Pi-starvation, the deprivation of other major nutrients does not stop the cell cycle at a specific phase, although cell growth is arrested as in the case of Pi depletion (Sano et al., 2004). Therefore, in the present study we tested whether the induction of autophagy under conditions of deprivation of different nutrients would also interact with each other early in the process, or whether there are specific sensing steps for each nutrient in the cell.

We showed previously that a protein aggregate formed of the fusion protein of Cyt b5 and RFP (Cyt b5-RFP) was a good substrate for autophagy in tobacco BY-2 cells (Toyooka et al., 2006). Because the intact and processed forms of Cyt b5-RFP can be distinguished easily after the separation of proteins by SDS–PAGE, quantification of the fluorescent intensities of RFP-related polypeptides can be used to calculate the efficiency of autophagy (Toyooka et al., 2006). However, this method of quantification has a limitation in that the reporter proteins produced before the induction of autophagy cannot be distinguished from those produced afterwards. In other words, this method cannot distinguish whether the small amounts of intact Cyt b5-RFP that can be detected in cells undergoing induced autophagy arise from the *de novo* synthesis of the reporter protein or via inefficient degradation of the protein. To overcome this problem, we improved the previously reported method by using a photoconvertible and tetrameric fluorescent protein, kikume green-red (KikGR; Tsutsui et al., 2005) as a substitute for RFP, and the results of the analysis of Pi-starvation-induced autophagy using this new reporter protein are described below.

## Materials and methods

The culture of tobacco BY-2 cells and transformation of this cell line were performed as described previously (http://mrg.psc.riken.go.jp/strc/BY-2tran.htm). The induction of autophagy using a medium deficient in Pi, nitrogen, or sugar was carried out as described previously (Toyooka et al., 2006). In some cases, dipotassium hydrogen phosphite (Phi; Kanto Kagaku Co. Inc., Tokyo, Japan) was included at a final concentration of 2.6 mM.

Construction of pMAT137-Cytb5-cKikGR, which is an expression plasmid for the Cyt b5-KikGR fusion protein under the enhancer-duplicated CaMV35S promoter, was done as follows. A fragment containing restriction enzyme digestion sites for KpnI at the 5' terminus and for ClaI at the 3' terminus of the KikGR-coding region was amplified by polymerase chain reaction (PCR) using pKikGR1-MC1 (MBL, Nagoya, Japan) as a template and TTTAGGTACCCATGGTGAGTGTGATTACAT and TTATATCGATTTACTTGGCCAGCCTTGGCA as primers. The resulting DNA fragment was digested with ClaI and KpnI and cloned into the corresponding sites of a binary expression plasmid pMAT137 (Yuasa et al., 2005) to yield pMAT137-cKikume. A DNA fragment encoding *Arabidopsis* Cyt b5 (At5g48810) was amplified by PCR using GCGGAGATCTGTCACCAGCAGATCATCGGAGATGGG and GGCCGGTACCAAGAAGAAGGAGCCTTGGTCTTAGTGTAGT as primers and a plasmid for the expression of Cyt b5-RFP (Toyooka et al., 2006) as a template. The resulting DNA fragment was digested using BglII and KpnI, and subcloned into the corresponding sites of pMAT137-cKikume to yield pMAT137-Cyt b5-cKikGR. Expression of NtAtg8-YFP in cells expressing Cyt b5-KikGR was carried out by transforming the Cyt b5-KikGR-expressing cells with *Agrobacterium* harboring the expression plasmid for NtAtg8-YFP (Toyooka et al., 2006).

Conversion of the fluorescence of Cyt b5-KikGR was carried out essentially as described previously (Abiodun and Matsuoka, 2013a) with a minor modification. In brief, a 100 ml aliquot of the cultured transformed cells at the exponential phase of growth in a 300 ml Erlenmeyer flask was exposed to purple light from a 100 W black light bulb (H100BL-L; Toshiba, Tokyo, Japan), which emits line spectra at wavelengths of 334, 365, and 404 nm, with shaking at room temperature for appropriate times.

Proteins were extracted from the cells as follows. After incubation in various media, cells were collected from cell suspension by centrifugation at 360 × *g* for 1 min in a 1.5 ml microfuge tube. Precipitated cells were suspended into approximately 10 volumes of phosphate-buffered saline (PBS; 4.3 mM Na_2_HPO_4_, 1.4 mM KH_2_PO_4_, 2.7 mM KCl, 137 mM NaCl, pH 7.4) and centrifuged as above. The resulting cell pellet was suspended with an equal volume of PBS and disrupted by sonication in an ice-cold water bath using a Bioruptor UCD-200TM sonicator (Cosmo Bio. Co. Ltd. Tokyo, Japan) at an M power setting for 1 min with 30 s interval 10 times. After centrifuging the microfuge tube at 360 × *g* for 5 min, the supernatant was collected and used for total protein fraction. Proteins were quantified using Bio-Rad Protein Assay kits (Bio-Rad Co., Hercules, CA, USA) as indicated by the manufacturer's instructions, using bovine serum albumin as a standard. To estimate the cell volume in culture, a 10-ml aliquot was centrifuged in a conical tube with graduations at 360 × *g* for 5 min and the volumes of cell precipitates were recorded. To weigh the cells, a 100 ml aliquot of the culture was filtered through a filter paper attached to a Buchner funnel with vacuum applied, and the weights of trapped cells were measured using a balance.

For detecting red and green fluorescence, aliquots of total protein fractions were mixed with 0.25 volumes of 5 × SDS sample buffer (250 mM Tris-HCl, pH 6.8, 50% (w/v) glycerol, 10% (w/v) SDS, 0.2 M dithiothreitol (DTT), 1% (w/v) bromophenol blue), and subjected directly to 9% SDS–PAGE without heating. After the separation of proteins by electrophoresis, the green and red fluorescence of proteins in the gels were recorded using a Typhoon 9400 image analyzer (GE Healthcare) with the following conditions: a 526 SP Cy2 filter set with a 488 nm laser running at 650 V was used for the recording of green fluorescence and a 580 BP30 Cy3 filter set with 532 nm laser running at 650 V was used for the recording of red fluorescence. The intensity of fluorescence was quantified using Image Quant Software (GE Healthcare).

For collecting epifluorescence images, an Olympus IX50 microscope equipped with DP70 color CCD camera (Olympus, Tokyo, Japan) was used. All the images were collected using a 20 × LC PLAN FL lens (Olympus). For the observation of green fluorescence, WIB filter/dichroic mirror cube (Olympus) was used. An RFP filter/dichroic mirror cube (Olympus) was used for detecting red fluorescence.

For collecting confocal images, a Leica TCS SP8 confocal microscope system (Leica Microsystems, Mannheim, Germany) equipped with a white light laser and HyD detectors was used. Images were captured using an HCPL APO CS2 40 × 1.30 oil lens with a pinhole of 44.1 μm of the confocal unit at an image resolution of 1024 × 1024 pixels at 100 Hz. For detecting green fluorescence, the excitation wavelength was 505 nm and signals of 514–546 nm were recorded using a HyD detector at a gain of 100. For detecting red fluorescence, the excitation wavelength was 555 nm and signals of 610–653 nm were recorded using a HyD detector at gain 101. At the same time, transmission images were recorded using a photomultiplier tube (PMT)-type detector. Only a single scan of each color using the line scan mode was used to collect each image.

The isolation of vacuole-enriched fractions from BY-2 cells and *in vitro* processing of reporter proteins were carried out essentially as described previously (Toyooka et al., 2006), except that the buffer conditions were changed as follows: the pH 6.6 and 6.0 buffers consisted of 8:2 and 6:4 mixtures of 50 mM Hepes-KOH pH 7.4 and citrate-Na pH 5.0, respectively.

## Results

### Generation of protein aggregates with a photoconvertible fluorescent protein in tobacco BY-2 cells and degradation under nutrient-starvation conditions

**Figure 1:**
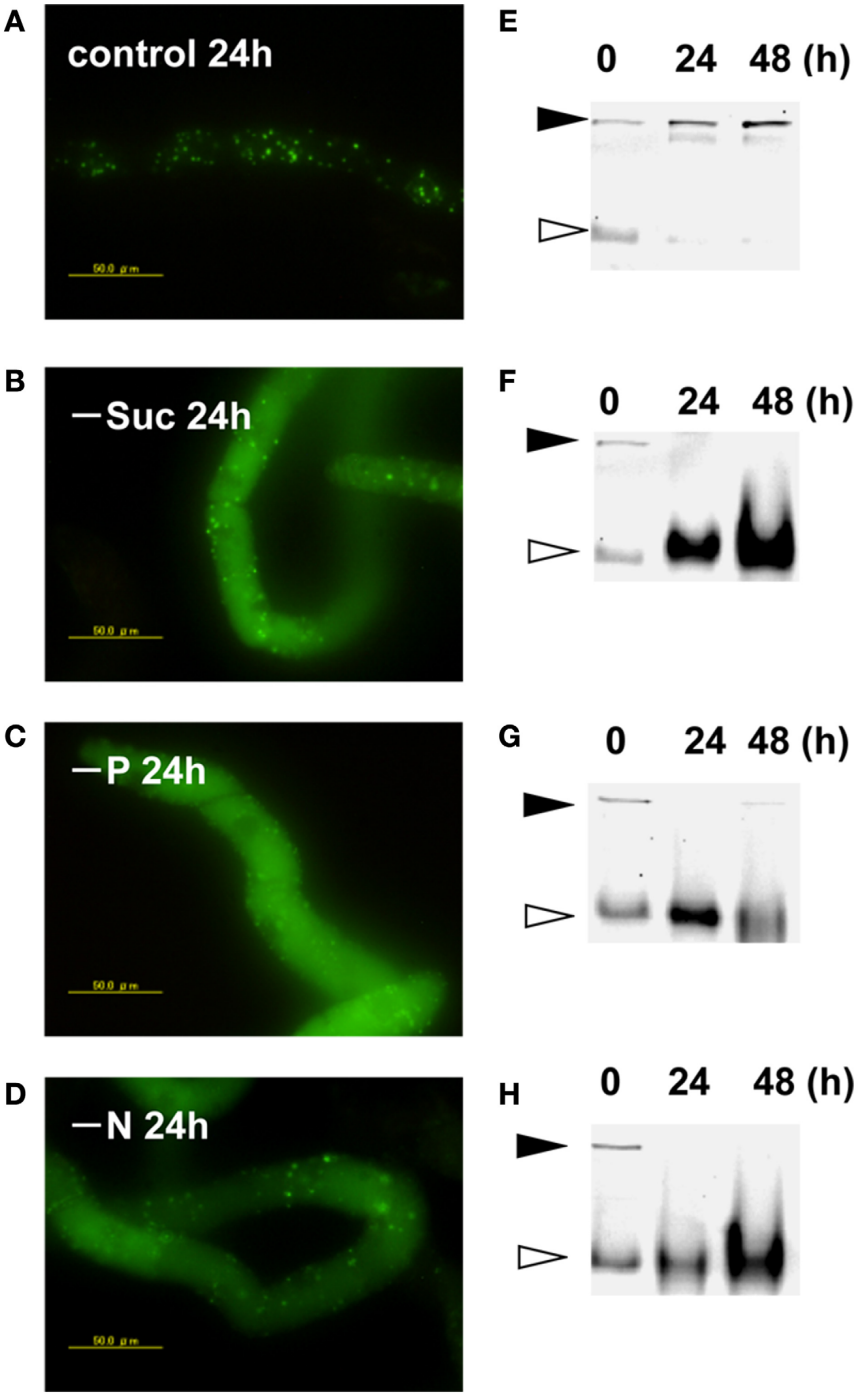
**Cytb5–KikGR aggregates were degraded by nutrient deprivation-induced autophagy in tobacco BY-2 cells**. Transformed tobacco BY-2 cells expressing Cyt b5-KikGR at the exponential growth phase were incubated with normal control medium **(A,E)**, sucrose-free medium **(B,F)**, phosphate (Pi)-free medium **(C,G)**, or nitrogen-free medium **(D,H)** and further cultured for 24 or 48 h. The epifluorescence image at 24 h **(A–D)** and migration pattern of KikGR fluorescent proteins on SDS–PAGE **(E–H)** at 0, 24, and 48 h of culture are shown. Each lane contains 5 μ g of protein from cell extracts. Black and white arrowheads indicate the migration positions of the intact and processed forms of Cyt b5-KikGR, respectively.

Transformed BY-2 cells expressing a fusion protein of Cyt b5 and KikGR (Cyt b5-KikGR) at the log-phase of growth contained protein aggregates of green fluorescence, but with little fluorescence in vacuoles. Many of the aggregates were spherical at 0.5–2 μm in diameter, similar to what we observed for Cyt b5-RFP aggregates (Toyooka et al., 2006). After the incubation of such cells in a normal medium for 24 h, the patterns of fluorescence were not changed significantly and only green puncta were found in the cells (Figure 1A). When cells in the log phase of growth were incubated in medium devoid of either sucrose, Pi, or nitrogen sources for 24 h, they showed strong fluorescence in the vacuoles (Figures 1B–D). Time-course analyses of the processing of the fusion protein under such starvation conditions revealed that the migration position of the fusion protein in SDS–PAGE changed from the intact form of approximately 160 kDa to a smaller form of approximately 100 kDa (Figures 1E–H). These behaviors of Cyt b5-KikGR in tobacco BY-2 cells were essentially identical to that of Cyt b5-RFP in the same cell line (Toyooka et al., 2006). Therefore, we concluded that the protein aggregates generated by Cyt b5-KikGR behave as suitable substrates for autophagy, as in the case of Cyt b5-RFP in tobacco BY-2 cells.

### Color conversion of the CYT b5-KikGR aggregate

**Figure 2:**
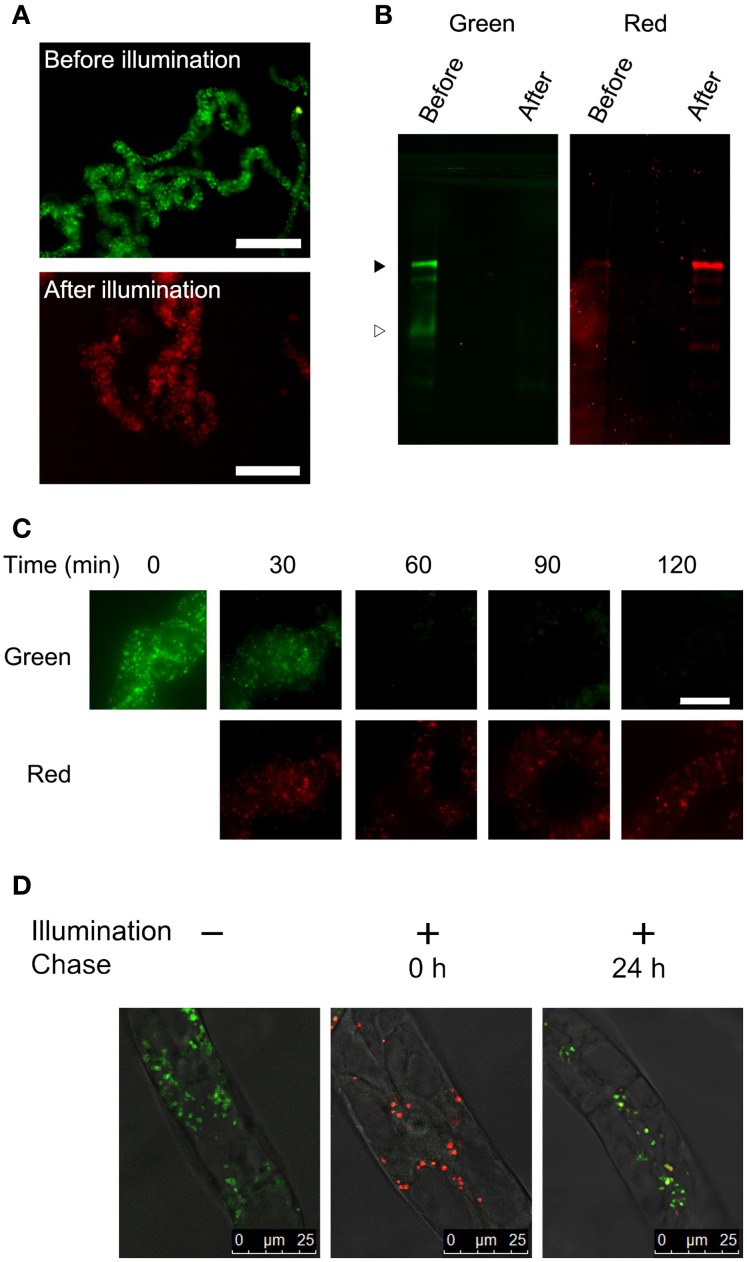
**Color conversion and the fate of aggregates under normal conditions**. **(A)** Low magnification epifluorescence images of cells before and after color conversion. Epifluorescence images of cells before and 1 h after illumination of purple light were collected using Olympus WIB and RFP filter/dichroic mirror sets. Bar = 200 μm. **(B)** Green and red fluorescence of proteins extracted from the transformant. Proteins extracted from the cells before and after illumination with purple light for 1 h were separated by SDS–PAGE, and green and red fluorescence images were recorded. Black and white arrowheads indicate the migration positions of the intact and processed forms of Cyt b5-KikGR, respectively. Each lane contained proteins corresponding to equal volumes of cells. **(C)** Time-course analysis of the conversion of fluorescence emission. The cell culture was illuminated with purple light at the indicated times and red and green fluorescence images were recorded using an epifluorescence microscope. Bar = 50 μm. **(D)** Analysis of the proliferation and *de novo* generation of the aggregates. Confocal fluorescence microscopic images are shown of Cyt b5-KikGR-expressing cells before purple light illumination, just after illumination for 1 and 24 h after illumination. Green and red fluorescence images as well as the transmission images were collected as indicated in the Materials and methods. Merged images are shown.

We have reported that fluorescence of a Golgi-targeted fusion protein comprising of a prolyl-hydroxylase NtP4H1.1 and monomeric KikGR could be converted under illumination by purple light, and this conversion allowed us to monitor the proliferation of the Golgi apparatus in tobacco BY-2 cells (Abiodun and Matsuoka, 2013a,b). We used the same conversion apparatus (Abiodun and Matsuoka, 2013a) to convert the fluorescence of the color of the aggregate from green to red. Three-day-old (mid-log phase) cells were illuminated with purple light and the fluorescence was monitored using an epifluorescence microscope. Within 1 h of illumination, nearly all the aggregates of Cyt b5-KikGR emitted red fluorescence (Figure 2A). Proteins extracted from the cells before and after illumination were separated by SDS–PAGE and their green and red fluorescence were recorded (Figure 2B). Before illumination with purple light, a major band of the intact-size Cyt b5-KikGR and a weak band of the processed form were detected by recording the green fluorescence. The red fluorescence recording allowed us to detect a faint band corresponding to intact Cyt b5-KikGR. After illumination, the green bands disappeared almost completely and bands with red fluorescence appeared. Time-course analysis of the fluorescence of aggregates in the cell indicated that the green fluorescence disappeared almost completely within 1 h of illumination (Figure 2C). These observations indicated that 1 h was sufficient for a near-complete conversion of the fluorescence color of Cyt b5-KikGR expressed in tobacco BY-2 cells.

Next, we analyzed whether the newly synthesized Cyt b5-KikGR protein would be incorporated into preexisting aggregates. We recorded both green and red fluorescence confocal images before and after color conversion. The color-converted cells were further grown for 24 h and confocal images of both green and red fluorescence were recorded. The merged images of the green and red fluorescence after 24 h incubation showed ring, dot, and line structures of varied colors from reddish orange to yellow and green (Figure 2D). Colors of many aggregates were not uniform and some parts of such aggregates were redder than others. These observations suggested that some of the newly synthesized Cyt b5-KikGR was incorporated into preexisting aggregates and that some other fraction contributed to formation of the new aggregate during the 24 h incubation period.

### Protein synthesis during starvation-induced autophagy

**Figure 3:**
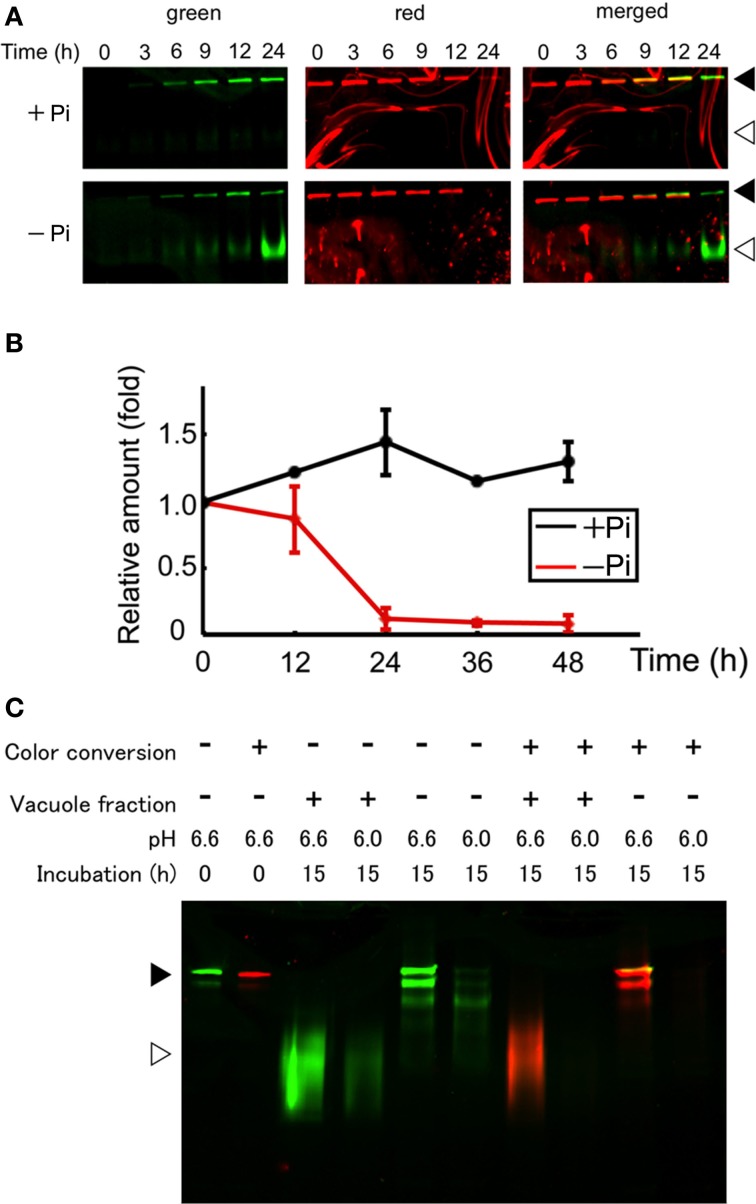
**Protein synthesis and autophagic degradation under phosphate-limited conditions**. **(A)** Short time-course analysis. Color-converted cells were re-suspended into normal (+Pi) and Pi-free (−Pi) medium and further cultured up to 24 h. Equal amounts of proteins (5 μ g) from the cells were separated by SDS–PAGE and fluorescence in the gel was recorded. Black and white arrowheads indicate the migration position of intact and processed forms of Cyt b5-KikGR, respectively. **(B)** Decrease of red-converted intact-size Cyt b5-KikGR during incubation. Cells were treated as described for panel **(A)**, and at each time point, the volume of cells in culture was measured. After the extraction of proteins from cells at the indicated time points, an equal amount (5 μ g) of protein was loaded to 9% SDS–PAGE and the intensity of the red-converted intact forms of Cyt b5-KikGR was quantified from the scanned image of the gel. Thereafter the amount of red-converted protein relative to that at the start of the experiment was calculated using the cell volume, the concentration of extracted protein, the volume of the extract, and the intensity of the protein band. The average of triplicated experiments is shown. The bar represents the SD. **(C)** *In vitro* fluorescence loss and processing with vacuole proteins of nonconverted and color-converted Cyt b5-KikGR under different pH conditions. Pellets of cell lysates from nonconverted or color-converted cells after 1000 × *g* centrifugation were used as the source of Cyt b5-KikGR aggregates. After incubation for the indicated times with buffers at the indicated pH, with or without vacuole-enriched fractions prepared from nontransformed cells cultured in Pi-deficient medium, the proteins were separated by SDS–PAGE and their emissions of green or red fluorescence were recorded. A merged image of the green and red fluorescence is shown. Black and white arrowheads indicate the migration position of the intact and processed forms of Cyt b5-KikGR, respectively.

After conversion of the fluorescence color of the aggregates, cells were washed and suspended in fresh medium with or without Pi and incubated further. The cells were harvested from an aliquot of the culture and proteins were extracted from the cells. Then, equal amounts of the proteins were separated by SDS–PAGE and the green and red fluorescence bands were recorded. Within 3 h after the start of incubation in normal medium, a green band of intact Cyt b5-KikGR was observed, and further incubation for up to 24 h increased its intensity (Figure 3A). In contrast, both intact and processed polypeptides emitting green fluorescence were detected after 3 h of incubation in cells under Pi-free conditions (Figure 3A). The intensity of the red fluorescence of the intact Cyt b5-KikGR decreased 24 h after color conversion in both media. No clear processed form of red fluorescence was observed in either case. The decrease in red fluorescence bands in cells after incubation might have arisen either from the dilution of preexisting protein during cell growth or degradation by autophagy, or both. To determine which of these systems contributed to the decrease, we carried out quantitative time-course analysis up to 48 h by measuring both the decrease in red fluorescence as well as cell growth, and estimated the relative amount of red-converted intact Cyt b5-KikGR in the net culture over time (Figure 3B). In the presence of Pi, the net amount of red-converted Cyt b5-KikGR per culture volume did not decrease up to 48 h of incubation. In the absence of Pi, the protein decreased almost completely after 24 h. This observation, as well as the formation of the green processed form under Pi-starvation, suggested that both preexisting and newly synthesized Cyt b5-KikGR were degraded by autophagy during the incubation.

Unlike the nonconverted (green) Cyt b5-KikGR, red-converted Cyt b5-KikGR did not yield clear processed bands under this starvation condition. To test whether this was the result of transport to the vacuoles, we analyzed whether processing by proteases in the vacuoles would eliminate the fluorescence of the red-converted processed form. We prepared sediment fractions from cell lysates of both native and photoconverted tobacco cells expressing Cyt b5-KikGR, and used them as a source of nonprocessed Cyt b5-KikGR protein aggregates. These fractions were incubated with buffers at either pH 6.6 or 6.0 with or without a vacuole-enriched fraction, which was prepared from nontransformed tobacco cells cultured in Pi-free medium. Thereafter, the proteins were separated by SDS–PAGE and the fluorescence was recorded (Figure 3C). In the presence of vacuoles, almost all the nonconverted Cyt b5-KikGR was converted into the fast-migrating form. However, such conversion was not detectable in the absence of the vacuole fraction, although a partially processed form that migrated to a slightly distant position from the sample well was observed, as in the case of Cyt b5-RFP (Toyooka et al., 2006). The green fluorescence of all bands was weaker at pH 6.0 than that at pH 6.6. When incubation was carried out using the sediment fraction from illuminated cells, the processing pattern at pH 6.6 was almost identical to that of the nonconverted one. In contrast, only a very slight amount of red fluorescence signal was observed at pH 6.0, regardless of the presence or absence of the vacuole fraction. These observations indicate that the red-converted fluorescence of Cyt b5-KikGR is more sensitive to acidic pH than that of the nonconverted one and suggest that our failure to detect the processed red fluorescent bands under Pi-free conditions was the result of autophagy-dependent vacuolar delivery of Cyt b5-KikGR, which caused denaturation of the fluorescence-converted KikGR protein under an acidic environment in the vacuoles.

### Phi retarded the induction of autophagy under Pi-starvation

**Figure 4:**
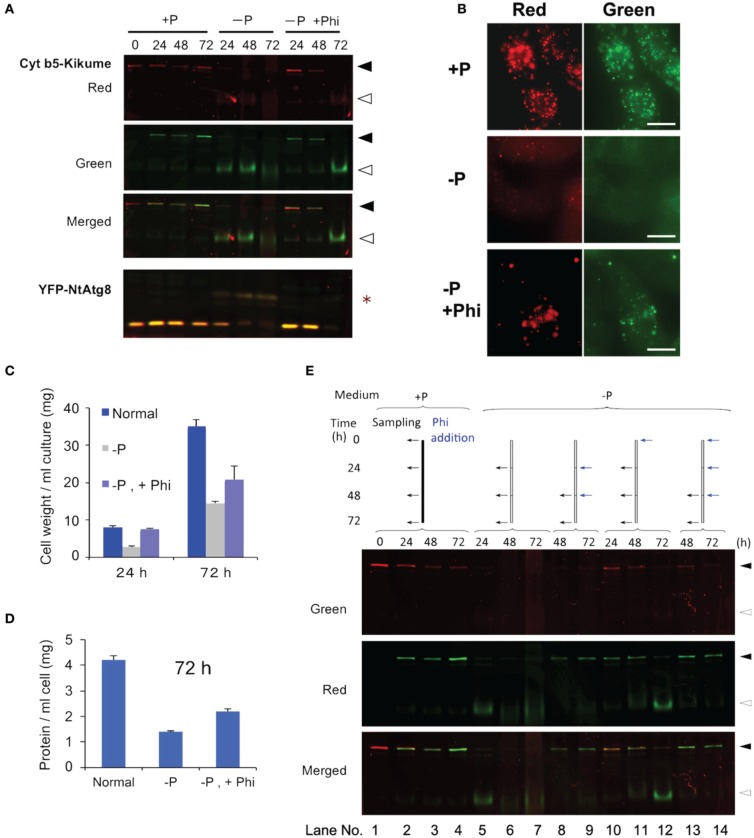
**Phosphite prevented Pi-starvation-induced autophagy**. **(A)** Time-course analysis of the effects of the presence or absence of Phi under Pi-free conditions. Cells expressing both Cyt b5-KikGR and YFP-NtAtg8 (Toyooka et al., 2006) were exposed to purple light, incubated in Pi-free medium with or without 2.6 mM Phi and cultured for the indicated times. Proteins extracted from the cells were analyzed as in the legend to Figure 3A. Black and white arrowheads indicate the migration positions of the intact and processed forms of Cyt b5-KikGR, respectively. Asterisk indicate the slow-migrating form of YFP-NtAtg8. **(B)** Epifluorescence images of cells incubated in normal (+P) or Pi-free (−P) medium with (+Phi) or without Phi for 24 h. **(C)** Effect of Phi on the growth of transformed tobacco cells under Pi-starvation. Transformed tobacco cells were incubated for the indicated times as described in panel **(A)** above, and the weights of cells were measured after removal of the culture medium by filtration. The average of triplicate experiments is shown and the bar represents the standard deviation (SD). **(D)** Effect of Phi on the amount of proteins in transformed tobacco cells under Pi-starvation. Transformed tobacco cells were incubated for 72 h as described in panel **(A)** above, and the amount of protein was measured after extraction. The mean of triplicate experiments is shown and the bar represents the SD. **(E)** Continued addition of Phi prevented the induction of autophagy. Color-converted cells expressing Cyt b5-KikGR were incubated in medium with or without Pi, and Phi was added to the culture to a final concentration of 2.6 mM. When multiple additions of Phi were carried out, the concentration of Phi at each time corresponded to an additional 2.6 mM in the medium. Culture conditions and the times of addition of extra lots of Phi were carried out as indicated at the top of the figure. Proteins extracted from the cells were analyzed as in the legend to Figure 3A. Black and white arrowheads indicate the migration positions of the intact and processed forms of Cyt b5-KikGR, respectively.

The Phi ion is a less oxidized form of phosphorous than the Pi ion and sometimes acts as a negative regulator of Pi deficiency-dependent gene expression in plants. Therefore, we tested whether Phi would prevent the induction of autophagy under Pi-starvation. Cells containing red-converted Cyt b5-KikGR aggregates were incubated in Pi-free medium with or without 2.6 mM Phi and cultured for up to 72 h. As shown in Figure 4A, the formation of the green processed form was prevented for up to 48 h in the presence of Phi. Decreases in red Cyt b5-KikGR were also prevented in the presence of Phi for up to 48 h. Under the same conditions, the Pi-starvation-dependent formation of the slow-migrating form of YFP-Atg8 as well as a decrease in YFP-Atg8 was also suppressed in the presence of Phi.

Microscopy of cells incubated under Pi-starvation for 24 h showed that vacuoles emitted faint red and green fluorescence with weak red fluorescence of aggregates (Figure 4B, −P). In the presence of Phi, red fluorescence in the vacuoles was not apparent and protein aggregates emit both red and green fluorescence (Figure 4B, −P, +P). This observation confirmed that the presence of Phi retarded the degradation of both preexisting and newly synthesized aggregates whereas newly synthesized Cyt b5-KikGR aggregates were degraded efficiently in the absence of Phi under Pi-starvation.

We also tested whether the suppression of cell growth subjected to Pi-starvation was restored by adding Phi. As shown in Figure 4C, an increase in cell volume was prevented under the Pi-starvation, and this was partially prevented by Phi. Likewise, the decreases in the concentrations of proteins in the cells subjected to Pi-starvation were partially prevented by Phi (Figure 4D). These observations suggest that Phi prevents several responses to Pi-starvation including the induction of autophagy in tobacco BY-2 cells.

Interestingly, the suppressive effect of Phi on the induction of autophagy did not continue to 72 h because the processed form of the nonconverted Cyt b5-KikGR was detectable at this time (Figure 4A, −P, +Phi, 72 h). In a previous study, it was reported that Phi was sequestered into the vacuoles of tobacco BY-2 cells within 48 h of its addition to the medium (Danova-Alt et al., 2008). Therefore, we tested whether the timing of the addition of Phi would affect the suppression of autophagy and the stability of preexisting (red) and newly synthesized (green) Cyt b5-KikGR (Figure 4E). When Phi was added 24 h after the shift to Pi-free medium and then the incubation was continued for 24 h (Figure 4E, lane 8), the level of intact Cyt b5-KikGR with red fluorescence was below the detection level and only green fluorescence was observed in the cells. Further addition of Phi and incubation for another 24 h did not change these results (Figure 4E, lane 9). In contrast, when Phi was included in the medium from the start and also augmented every 24 h, the preexisting Cyt b5-KikGR was still detectable after 72 h of incubation (Figure 4E, lane 14). This observation and the previous findings (Danova-Alt et al., 2008) suggest that the presence of Phi in the cytoplasm is necessary to suppress the induction of autophagy in tobacco BY-2 cells under Pi-starvation.

### Phi did not prevent induction of autophagy under sugar- or nitrogen-starvation

**Figure 5:**
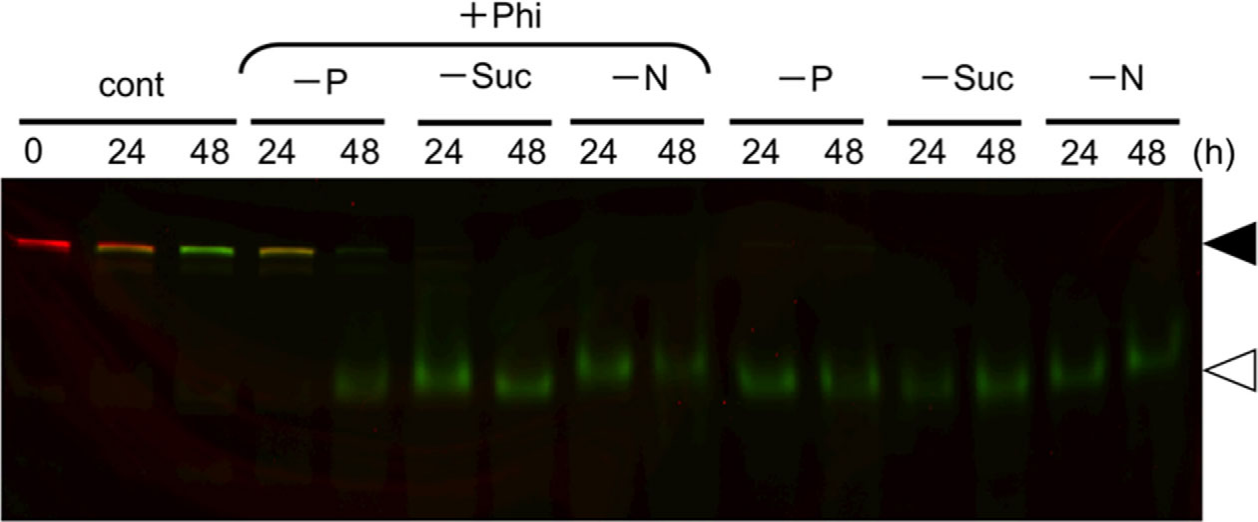
**Phosphite did not prevent the induction of autophagy under sucrose—or nitrogen-starvation**. Color-converted cells expressing Cyt b5-KikGR were incubated in medium with or without one of the nutrients as described in the legend to Figure 1A with or without 2.6 mM Phi for the indicated times, and analyzed as in the legend to Figure 3A. Only a color-merged image is shown. Black and white arrowheads indicate the migration positions of the intact and processed forms of Cyt b5-KikGR, respectively.

Starvation of carbon and nitrogen sources induces autophagy in tobacco BY-2 cells (Moriyasu and Ohsumi, 1996; Toyooka et al., 2006). It was also reported that, in some cases, the signaling pathways related to sugar- and Pi-responses interact with each other in plants (Rouached et al., 2010). Therefore, we tested whether Phi would also affect the induction of autophagy in the absence of these nutrients (Figure 5). We found that the presence of Phi did not prevent a decrease in the intact form of both red-converted and green Cyt b5-KikGR, and allowed the formation of processed green Cyt b5-KikGR. Thus, treatment with Phi had no effect on the induction of autophagy under sugar- or nitrogen-starvation.

## Discussion

We observed that the fusion protein of Cyt b5 and KikGR forms aggregates as in the case of the Cyt b5 and RFP fusion protein (Figure 1). The protein aggregates are able to change their fluorescence color after illumination with purple light, as in the case of the intact KikGR protein. The color-converted aggregates change color from red to orange, yellow or both red and green after the incubation of cells in normal medium (Figure 2D). This observation suggests that the aggregates are not static structures and can incorporate newly synthesized proteins. However, we cannot rule out the possibility that the formation of the aggregates with both green and red fluorescence were the result of *de novo* formation of newly synthesized (green) Cyt b5-KikGR and the preexisting (red-converted) Cyt b5-KikGR that had not contributed to generate aggregates. We also cannot rule out the possibility that some of the images of aggregates with both green and red colors (Figure 4D) were the result of close association of aggregates of two different colors because the resolution of our fluorescence microscope was not high enough. Future time-course analyses chasing one aggregate will be necessary to determine whether these aggregates with both colors were derived from color-converted aggregates.

Our observations here and our recent analysis of Golgi proliferation using a monomeric KikGR-tagged reporter protein (Abiodun and Matsuoka, 2013a,b) indicate that bulk conversion of the reporter fluorescence color and subsequent analysis is a method that can be applied to analyze the synthesis and/or turnover of distinct intracellular structures. However, this method has a limitation when used for the quantitative comparison of preexisting and newly synthesized proteins. The fluorescence of the red-converted KikGR is more sensitive to acidic pH than that of the nonconverted KikGR (Figure 3C). Despite this limitation, the method could be used successfully for the analysis of inducible protein turnover and/or intracellular movements. Analysis of protein turnover after separating the proteins by SDS–PAGE and subsequent scanning of both red and green fluorescence allowed the quantification of preexisting and newly synthesized proteins (Figure 3). However, such protein samples should be handled as quickly as possible because both sunlight and light from regular fluorescent lamps contain wavelengths that can cause the conversion of the fluorescence color of KikGR. The presence of faint red signals in nonconverted samples (Figure 2B) could have arisen from the conversion of fluorescence during sample processing.

The depletion of extracellular nutrients such as sugar, nitrogen, or Pi induced degradation of the aggregate. Thus, both the degradation of preexisting (red-converted) Cyt b5-KikGR and the synthesis of green-fluorescent Cyt b5-KikGR were observed in color-converted cells (Figure 3). The kinetics of the increase in green Cyt b5-KikGR were not significantly different from the Pi-limited and normal conditions up to 12 h (Figure 3A). Autophagic degradation was not observed early after the change in medium, and only a small decrease in color-converted protein was observed at 12 h after the shift to nutrient-starved medium. This suggested that the induction of autophagy under Pi-starvation was not a result of the sensing of the level of extracellular Pi, but rather that some intracellular events might be required. Protein synthesis was not attenuated at the time when autophagy was already induced (Figure 3A, at 24 and 48 h). This suggests that *de novo* protein synthesis might be taking place under Pi-starvation and autophagic degradation of the cellular constituents might supply energy and building blocks for protein synthesis.

Under Pi-starvation, we observed a decrease in a form of YFP-NtAtg8A found in nonstarved cells, and an increase in the slowly migrating form of YFP-NtAtg8 (Figure 3A). The formation of the slow-migrating form was suppressed in the presence of Phi when autophagic degradation of the aggregates was not occurring (Figure 3A). Although the nature of this form is unknown, similar slowly migrating forms of the Atg8 protein have been observed in *Arabidopsis*, especially in mutants deficient in Atg8/12 conjugation systems (Thompson et al., 2005; Chung et al., 2010). Interestingly, such forms were not found in maize (Chung et al., 2009). Therefore, it will be interesting to characterize this in terms of whether multiple orthologs of Atg8s in *Arabidopsis* and tobacco (Thompson et al., 2005; Toyooka and Matsuoka, 2006) generate such forms under different conditions of autophagic induction. It will also be interesting to investigate whether such processing is related to a plant-specific autophagy-related compartment that is generated under carbon-starvation (Honig et al., 2012).

The induction of autophagy was retarded when Phi was included in the Pi-deficient medium (Figure 3). Phi is a nonmetabolizable analog of Pi that does not support the growth of plants (Thao and Yamakawa, 2009). However, it has been shown to affect multiple events related to Pi metabolism. Thus, Phi attenuates several, but not all, *in planta* responses to Pi-starvation (Ticconi et al., 2001, 2004; Varadarajan et al., 2002; Kobayashi et al., 2006; Stefanovic et al., 2007; Ribot et al., 2008; Li et al., 2010; Berkowitz et al., 2013). Moreover, Phi mimics Pi in terms of the inducible degradation of Pi-deficient inducible phosphatase (Bozzoa et al., 2004), and Phi accelerates programmed cell death induced by Pi-starvation in cultured brassica cells (Singh et al., 2003). Our observations indicate that not only the *in planta* response to Pi deficiency, but also the induction of autophagy are prevented by Phi (Figure 4). However, the effect was lost at 72 h after incubation in Pi-free medium when Phi was included from the start. This limited effect can be explained by the observation that Phi rapidly absorbed from the medium is slowly sequestered into the vacuoles in tobacco BY-2 cells (Danova-Alt et al., 2008). In fact, subsequent addition of Phi under the Pi-deficient induction condition continued to prevent the induction of autophagy (Figure 4E). Thus, cytosolic Phi might mimic Pi and prevent the induction of autophagy.

Some of the signal transduction pathways induced by Pi deficiency merge with sugar-signaling pathways (Rouached et al., 2010 and references therein). However, our observation that Phi did not prevent the induction of autophagy in sugar-starved medium (Figure 5) suggests that the sensing mechanism for Pi-starvation that induces autophagy is independent of sugar signaling. Likewise, Phi did not prevent the induction of autophagy under nitrogen-starvation (Figure 5). In the case of sugar starvation-dependent induction of autophagy, it was reported in sycamore maple cells that the level of energy supply is crucial, and that the inclusion of pyruvate, which is a good substrate for the mitochondrial energy supply system, could suppress sugar starvation-inducible autophagy (Aubert et al., 1996). In mammalian and yeast systems, the target of rapamycin (TOR) protein kinase has been shown as a key negative regulator for nutrient limitation-induced autophagy (Díaz-Troya et al., 2008). It was also shown in *Arabidopsis* that constitutive autophagy is negatively regulated by TOR using an interfering RNA (RNAi)-based suppression approach (Liu and Bassham, 2010). Therefore, it would be interesting to apply the present approach, along with pyruvate and/or respiratory inhibitors or downregulation of the expression of TOR, to analyze whether the cellular mechanisms used to sense limitations in the supply of different nutrients and in the induction of autophagy might be associated.

### Conflict of interest statement

The authors declare that the research was conducted in the absence of any commercial or financial relationships that could be construed as a potential conflict of interest.
